# A Mosaic of Geothermal and Marine Features Shapes Microbial Community Structure on Deception Island Volcano, Antarctica

**DOI:** 10.3389/fmicb.2018.00899

**Published:** 2018-05-07

**Authors:** Amanda G. Bendia, Camila N. Signori, Diego C. Franco, Rubens T. D. Duarte, Brendan J. M. Bohannan, Vivian H. Pellizari

**Affiliations:** ^1^Departamento de Oceanografia Biológica, Instituto Oceanográfico, Universidade de São Paulo, São Paulo, Brazil; ^2^Department of Biology, Institute of Ecology and Evolution, University of Oregon, Eugene, OR, United States

**Keywords:** polar marine volcano, Antarctica, environmental gradients, extremophiles, diversity, community structure

## Abstract

Active volcanoes in Antarctica contrast with their predominantly cold surroundings, resulting in environmental conditions capable of selecting for versatile and extremely diverse microbial communities. This is especially true on Deception Island, where geothermal, marine, and polar environments combine to create an extraordinary range of environmental conditions. Our main goal in this study was to understand how microbial community structure is shaped by gradients of temperature, salinity, and geochemistry in polar marine volcanoes. Thereby, we collected surface sediment samples associated with fumaroles and glaciers at two sites on Deception, with temperatures ranging from 0 to 98°C. Sequencing of the 16S rRNA gene was performed to assess the composition and diversity of Bacteria and Archaea. Our results revealed that Deception harbors a combination of taxonomic groups commonly found both in cold and geothermal environments of continental Antarctica, and also groups normally identified at deep and shallow-sea hydrothermal vents, such as hyperthermophilic archaea. We observed a clear separation in microbial community structure across environmental gradients, suggesting that microbial community structure is strongly niche driven on Deception. Bacterial community structure was significantly associated with temperature, pH, salinity, and chemical composition; in contrast, archaeal community structure was strongly associated only with temperature. Our work suggests that Deception represents a peculiar “open-air” laboratory to elucidate central questions regarding molecular adaptability, microbial evolution, and biogeography of extremophiles in polar regions.

## Introduction

Despite its predominantly cold ecosystems, Antarctica harbors active volcanoes with versatile and extremely diverse microbial communities. These are unique habitats where psychrophiles, mesophiles, thermophiles, and hyperthermophiles coexist and interact in the same environment across a pronounced temperature gradient (Amenábar et al., 2013). At present, there are four active volcanoes in Antarctica, three located in continental sites, and one in maritime Antarctica (called Deception Island) (Kyle and Cole, 1974; Herbold et al., 2014b). Deception Island differs from continental volcanoes specifically by its strong marine influence and higher temperatures, reaching values of 100°C next to active fumaroles, while continental volcanoes reach values up to 65°C (Muñoz-Martín et al., 2005; Herbold et al., 2014b).

Deception Island is also notable for its varied and steep environmental gradients. Because over half of Deception Island is covered by glaciers, there are pronounced temperature gradients over very short distances (e.g., a few meters) (Baker, 1969; Bartolini et al., 2014). There can be strong salinity gradients from glaciers to fumaroles, because the fumaroles are located in the intertidal zone. In addition to temperature and salinity, there are prominent geochemical gradients generated by continuous emissions of volcanic gases, creating a mosaic of environmental conditions that can favor metabolically diverse microbial communities (Amenábar et al., 2013; Herbold et al., 2014b).

Little is known about how volcanic activity may influence microbial communities in polar ecosystems, despite extensive study of geothermal sites in nonpolar regions (e.g., Antranikian et al., 2017; Price and Giovannelli, 2017; Ward et al., 2017), and Deception Island offers a unique opportunity to better understand these important ecosystems. Because of the geographical isolation and the predominantly cold habitats in Antarctica, we expect that community structure may differ from those of nonpolar geothermal systems. Furthermore, the existence of multiple steep environmental gradients represents a unique opportunity to understand the drivers of microbial community structure and diversity in geothermal polar regions. Several studies have shown that microbial communities can be structured by environmental parameters such as temperature, salinity, and geochemistry (e.g., Crump et al., 2004; Sharp et al., 2014; Antranikian et al., 2017; Price and Giovannelli, 2017; Ward et al., 2017); however, it isn’t clear how these different drivers interact and simultaneously affect community structure.

To date, only two studies have used molecular methods to survey microbial community composition and diversity on Deception Island. These studies sampled Deception fumaroles, and characterized the microbial communities of these samples using denaturing gradient gel electrophoresis (DGGE) and subsequent sequencing of DGGE bands. These studies reported the presence of bacterial taxa, primarily members of the Firmicutes and Thermus/Deinococcus phyla, and observed for the first time hyperthermophilic Archaea in Antarctica. These studies were very limited, in both sampling extent (only fumaroles were sampled) and sampling depth (only relative coarse molecular techniques were used), and highlight the need for a more comprehensive survey of microbial communities on Deception Island (Muñoz et al., 2011; Amenábar et al., 2013).

In order to better understand how microbial community structure is shaped by gradients of temperature, salinity, and geochemistry in polar marine systems, we sampled sediments associated with fumaroles and glaciers from two geothermal sites on Deception Island, spanning temperatures between 0 and 98°C, and we used next-generation sequencing to characterize the communities in these samples. We observed that temperature, pH, salinity, and nutrient concentrations together explained significant amounts of the variation in bacterial diversity, whereas variation in archaeal diversity was primarily explained by temperature. Furthermore, we observed that these factors interacted to alter bacterial and archaeal community composition. Additionally, we observed the coexistence of putative psychrophiles, mesophiles, thermophiles, and hyperthermophiles, and that this unique community structure reflects the mosaic of environmental conditions created by interaction between the volcanic activity, the marine environment, and the cryosphere.

## Materials and Methods

### Study Site and Sampling Strategy

Deception Island (62°58′ S, 60°39′ W) is a complex, horseshoe-shaped stratovolcano whose central part collapsed during an eruption approximately 10,000 years ago, giving rise to a caldera called Port Foster Bay, approximately 9 km in diameter (Baker et al., 1975). Geothermal anomalies are found mainly at Fumarole Bay (FB), Whalers Bay (WB), and Pendulum Cove, probably originating during the last eruptions between 1967 and 1970 (Fermani et al., 2007). Fumaroles are distributed both in submerged and partially submerged regions (intertidal zones). Sediment temperature associated with fumaroles varies considerably, reaching values between 40 and 60°C in WB, 70°C in Pendulum Cove, and 80 and 100°C in FB (Rey et al., 1995; Somoza et al., 2004). Fumarolic gases in Deception are mainly composed by CO_2_ and H_2_S (Somoza et al., 2004), which in contact with atmospheric O_2_ is oxidized to products as sulfite ($SO_{3}^{2-}$) and sulfate ($SO_{4}^{2-}$) (Zhang and Millero, 1993).

**FIGURE 1:**
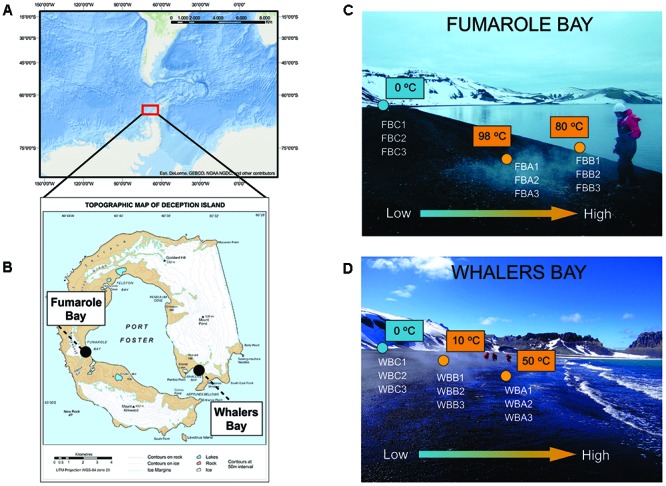
Sampling map. Location of Antarctic Peninsula **(A)** and Deception Island, with Fumarole Bay and Whalers Bay (WB) geothermal sites highlighted **(B)**. The map in **(A)** was generated using ESRI ArcGIS software. The map in **(B)** was courtesy of British Antarctic Survey. Distribution of collected samples across environmental gradients at studied geothermal sites is described in **(C)** for FB and **(D)** for WB. Values of *in situ* temperatures are represented in blue (glaciers) and orange (fumaroles). The arrow indicates the direction of low and high values of temperature, salinity, and volcanic compounds, as sulfate.

Sampling was performed during the XXXII Brazilian Antarctic Expedition (December 2013–January 2014), with logistical support from the polar vessel Npo. Almirante Maximiano. Surface sediment samples (ca. 5 cm) were collected in fumaroles and glaciers at the geothermally active sites of FB (62°58′02.7′′ S, 60°42′36.4′′ W) and WB (62°58′45.1′′ S, 60°33′27.3′′ W) (**Figures 1A,B**). In each site, three sediment samples were collected in each of three points with distinct temperatures: Points A and B were defined as samples collected in fumaroles, while point C was glacier samples, collected below the glacier’s edge (**Figures 1C,D**). Distances between fumaroles and glaciers at each site were approximately 15 m, and the WB and FB transects were approximately 10 km apart. All fumaroles were in the intertidal zone, with exception of point B from FB, which was in the subtidal (submerged at 50 cm depth in water column). Samples were stored at -20°C until arrival at the University of São Paulo, Brazil, in April 2014.

### Physicochemical Analysis

We evaluated physicochemical parameters of the sediments, including granulometry, electrical conductivity, humidity, micronutrients (B, Cu, Fe, Mn, and Zn), organic matter, organic carbon, pH, P, Si, Na, K, Ca, Mg, Al, total nitrogen, nitrate, ammonia, and sulfate. These analyses were conducted at “Luiz de Queiroz” College of Agriculture (Department of Soil Sciences, ESALQ-USP, Brazil), according to methods previously described (Keeney and Nelson, 1982; Van Raij et al., 2001).

### DNA Extraction and 16S rRNA Gene Sequencing

Total genomic DNA was extracted from 10 g of sediment using a PowerMax Soil DNA Kit (MoBio, United States), according to the manufacturer’s protocol. Extracted DNA was concentrated and purified with PCR OneStep Inhibitor Removal Kit (Zymo Research, United States), and further quantified using Qubit dsDNA HS Assay (Thermo-Fisher Scientific, United States) and Qubit Fluorimeter 1.0 (Thermo-Fisher Scientific, United States). Microbial 16S rRNA gene fragments were amplified using the primers S-D-Bact-0341-b-S-17 and S-D-Bact-0785-a-A-21 for Bacteria, and S-D-Arch-0519-a-S-15 and S-D-Arch-1041-a-A-18 for Archaea (Klindworth et al., 2013), targeting the V3–V4 regions of the gene. The first PCR reaction was carried out with a thermal cycler (Thermo-Fisher Scientific, United States), using 25 μL of KAPA HiFi HotStart Ready Mix (KAPA Biosystems) polymerase, 5 ng of DNA, and 0.2 μM of each primer, under the following conditions: 95°C for 3 min, 30 cycles of 95°C for 30 s, 55 or 67°C for 30 s (for Bacteria and Archaea, respectively), 72°C for 30 s, and a final extension of 72°C for 5 min. After purification (QIAquick Gel Extraction Kit – QIAGEN, United States) and quantification, 50 ng of amplicons was amplified and used for library preparation, under the following conditions: 95°C for 3 min, eight cycles of 95°C for 30 s, 55 and 72°C for 30 s, and 72°C for 5 min. The libraries were purified using an AMPure XP beads kit (Beckman Coulter, United States). After quality checking (Bioanalyzer 2100, Agilent Technologies, United States), the amplicons from each sample were mixed at equimolar concentrations and then sequenced using the Illumina Miseq platform at the Facilities Center for Research Support (CEFAP, Institute of Biomedical Sciences, University of São Paulo).

### Sequencing Data Processing and Statistical Analyses

Raw sequencing reads were filtered for length (>400 bp), quality score (mean, >30), and minimum expected errors of 1.0 using USEARCH tools (Edgar, 2010) and PRINSEQ Software (Schmieder and Edwards, 2011). Paired-end reads were assembled using PEAR software (Zhang et al., 2014), with a minimum overlap of 50 bp. Sequences were clustered at 97% similarity using USEARCH (Edgar, 2010) including *de novo* and reference-based chimera checking (ChimeraSlayer) (Haas et al., 2011). Operational taxonomic units (OTUs) with singletons (*n* = 1) were removed. Taxonomy was assigned to each OTU by performing BLAST searches against the Silva database v. 132 (updated on December 2017) (Quast et al., 2013), with a maximum *E*-value of 1e-5. Sequences were filtered for only bacterial or archaeal sequences for further analyses in the Quantitative Insights into Microbial Ecology (QIIME) 1.8.0 pipeline (Caporaso et al., 2010). The phylogenetic tree was built using FastTree (Price et al., 2009). Alpha-diversity indexes (number of OTUs, Ace richness estimation, and Shannon and Simpson) were calculated, and differences in alpha-diversity estimates between groups of samples were tested using Student’s *t*-test in R. OTU table was normalized for beta-diversity analysis, using cumulative sum scaling – CSS (Paulson et al., 2013). Beta-diversity between samples was examined using a Bray–Curtis dissimilarity matrix visualized as a UPGMA dendrogram, as well as by weighted normalized Unifrac distance, visualized via non-metric multidimensional scaling (nMDS), with fitting of the environmental parameters (only parameters with *p* < 0.05 were represented), accomplished with the *envfit* function from the *vegan* package (Oksanen et al., 2013). To test the significance of differences between groups of samples (Fumaroles vs. Glaciers and FB vs. WB), analysis of similarity (adonis) using Unifrac values was performed. We performed Spearman correlations to determine relationships between community composition (selecting abundant OTUs, with >1% of relative abundance) and environmental parameters. Only parameters that exhibited *p* < 0.05 with at least one OTU were represented. In order to evaluate which combination of parameters were related to alpha-diversity (Shannon index), we performed multiple linear regressions. We have applied univariate linear regressions to select only the significant parameters (*p* < 0.05) for multiple linear regressions. All sequencing data were deposited in the National Center for Biotechnology Information Sequence Read Archives (SRA) under BioProject ID PRJNA386506. Graphs and statistical analysis were carried out using R software (version 3.3.1), and the packages *ggplot2*, *vegan*, *qiimer*, *reshape2*, and *flyr*.

## Results

### Physicochemical Characteristics of the Sampling Site

Temperature measured *in situ* varied from 0 to 98°C. Fumarole temperatures were 50°C (WBA1, WBA2, and WBA3) and 10°C (WBB1, WBB2, and WBB3) for WB, and 98°C (FBA1, FBA2, and FBA3) and 80°C (FBB1, FBB2, and FBB3) for FB. FB and WB glaciers exhibited temperatures near 0°C. Values of pH varied between 6 (WBC3) and 7.9 (FBC2) for glaciers, and from 6.7 (WBA2 and FBA1) to 7.4 (WBB2) for fumaroles. Sediments were mainly composed of sand (representing 61.9–96.7% of sediment composition) (**Supplementary Table S1**).

Samples from the WB glaciers were characterized by higher concentrations of nitrogen compounds, as ammonia (53–150 mg kg^-1^) and nitrate (18–206 mg kg^-1^), when compared to the fumaroles (ammonia: 11–70 mg kg^-1^; nitrate: 11–84 mg kg^-1^). Samples from the WB glacier exhibited the highest concentrations of total nitrogen (1344–1421 mg kg^-1^) in comparison to the other samples (280–567 mg kg^-1^). By contrast, fumarole samples exhibited higher concentrations of marine (Na: 15.1–144.1 mmolc kg^-1^; electrical conductivity: 429–6595 μS cm^-1^) and volcanic geochemicals (sulfate: 125–293 mg dm^-3^), when compared to the glaciers (Na: 3.4–12.9 mmolc kg^-1^; electrical conductivity: 84–210 μS cm^-1^; sulfate: 4–9 mg dm^-3^). Concentrations of Fe were higher in FB fumaroles (40–296 mg dm^-3^) in comparison to WB fumaroles (23–72 mg dm^-3^) and glaciers (45–115 mg dm^-3^).

### Prevalent Taxa in Deception Island Glaciers vs. Fumaroles

In this study, we used bacterial and archaeal 16S rRNA primer sets to obtain a total of 1,700,412 and 1,684,699 high-quality reads, respectively, from 18 sediment samples of fumaroles and glaciers. A total of 5,884 OTUs ranging from 706 (FBB1) to 1,868 (FBC1) OTUs per sample were classified as Bacteria, whereas a total of 120 OTUs ranging between 4 (FBA2) and 44 (FBC1) OTUs per sample were assigned as Archaea (**Supplementary Table S2**). Even after several efforts, samples from FB fumarole at 98°C (FBA1, FBA2, and FBA3) could not be amplified for Bacteria in the analyzed conditions. In addition, no archaeal sequences were detected in samples from the WB glacier (WBC1, WBC2, and WBC3). Quantitative PCR analysis was performed by our group with the same primers here employed and showed a very low abundance of Archaea in WB glacier (unpublished data).

**FIGURE 2:**
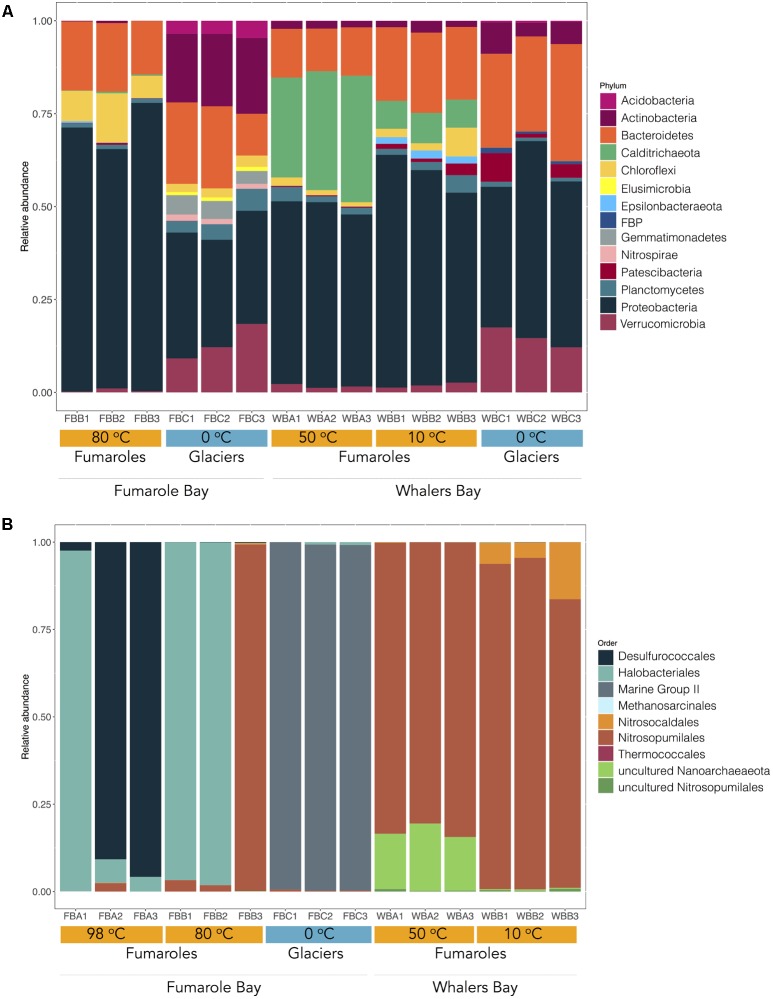
Microbial community composition in fumaroles (orange) and glaciers (blue) on Deception Island. The figure shows the relative abundance of bacterial **(A)** and archaeal **(B)** taxonomic groups at phylum and order levels, respectively. Only bacterial phyla with more than 0.1% of abundance are represented. Environmental temperatures and geothermal sites of each sample are represented. Sequences were clustered at 97% similarity and taxonomy was assigned by performing BLAST searches against the Silva database v. 132 (*E*-value ≤ 1*e–*5).

Looking at phylum level, some bacterial groups were common across our samples (**Figure 2A**). For example, the bacterial groups Proteobacteria (Gammaproteobacteria and Alphaproteobacteria class), Planctomycetes (class Planctomycetacia, order Pirellulales), and Bacteroidetes (class Bacteroidia, order Flavobacteriales) were abundant in all glaciers and fumaroles (except FBA1, FBA2, and FBA3) analyzed in this study. Proteobacteria was the most abundant, comprising about 50% of the total taxonomic composition of each sample. Within Proteobacteria, Gammaproteobacteria (17.81.74–51.48%) was the most abundant class, followed by Alphaproteobacteria (10–27.78%).

**FIGURE 3:**
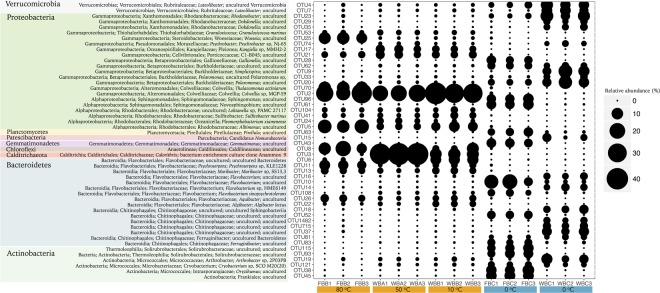
Classification of the 50 most abundant OTUs for Bacteria. The size of circles is related to the relative abundance of each OTU. OTUs are organized by phylum level.

**FIGURE 4:**
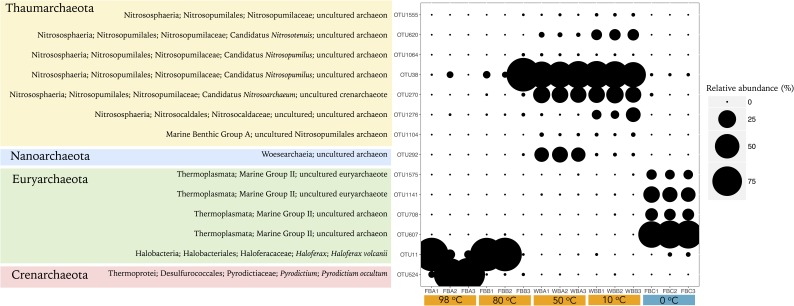
Classification of the most abundant OTUs for Archaea (>0.1% of relative abundance). The size of circles is related to the relative abundance of each OTU. OTUs are organized by phylum level.

Most taxonomic groups varied in abundance or occurrence between glacier and fumarole samples even at phylum level. For example, WB and FB glaciers exhibited the highest relative abundance of the bacterial phyla Verrucomicrobia (9.15–18.41%), FBP (0.6–1.48%), Gemmatimonadetes (3.45–5.20%), Acidobacteria (0.3–4.6%), and Nitrospirae, in comparison to fumaroles (0.2–2.6, 0.0–0.1, 0.0, 0.0–0.2, and 1.3–1.7%, respectively). Prevalent genera in glaciers were *Flavobacterium*, *Luteolibacter*, *Rhodoferax*, *Rhodanobacter*, *Dokdonella*, and *Polaromonas* (**Figure 3**). The archaeal Thermoplasmata class (phylum Euryarchaeota) was dominant (>90%) in FB glacier samples, but not detected in fumarole samples (**Figure 2B**), and was exclusively represented by Marine Group II (**Figure 4**). When aligned with RDP database 11 (updated on 2016) (Cole et al., 2014), OTU1141 and OTU1575, related to archaeal Marine Group II, showed 85% of identify with *Methanomassiliicoccus* sequences.

Fumarole samples displayed the highest occurrence of Calditrichaeota (previously classified as Deferribacteres) and Chloroflexi bacterial phyla (**Figure 2A**). Calditrichaeota was abundant in WB fumarole samples, varying from 26.86 to 34.04% in WBA1, WBA2, and WBA3, and from 7.50 to 8.16% in WBB1, WBB2, and WBB3, and was less abundant in FB fumarole samples at 80°C (0.1–0.4%). Representatives of Calditrichaeota were not identified in glacier samples. Chloroflexi was identified in all samples associated with fumaroles (except for FB fumarole at 98°C) and had greater abundance in the FB fumarole at 80°C (5.98–13.24% for FBB1, FBB2, and FBB3). The most abundant classified genera of Bacteria in fumarole samples were related to Alphaproteobacteria (*Albimonas*, *Loktanella, Pleomorphobacterium*, and *Sulfitobacter*) and Gammaproteobacteria class (*Thalassomonas* and *Woeseia*), and Calditrichaeota phylum (*Calorithrix*, previously assigned as *Caldithrix*) (**Figure 3**). Sequences related to Chloroflexi phylum, Anaerolineae class, Caldilineaceae family were not assigned at genus level. Archaeal composition in fumaroles varied according to the temperature gradients, and no phylum was dominant among all samples. For example, samples from the WB fumaroles were dominated by Thaumarchaeota (>95%), mostly represented by Nitrosopumilales (80.5–94.9%) and Nitrosocaldales (4.4–16.3%). The classified genera within Nitrosopumilales were *Nitrosopumilus*, *Nitrosoarchaeum*, and *Nitrosotenuis* (**Figure 4**). Samples from the WB fumarole at 50°C (WBA1, WBA2, and WBA3) had approximately 20% of sequences classified as Nanoarchaeota phylum. Although one sample of FB fumarole of 80°C (FBB3) showed a similar archaeal composition to WB fumaroles (>90% of OTUs related to Nitrosopumilales order), the others (FBB1 and FBB2) were dominated by euryarchaeotal members related to *Haloferax* genus (98%), which was assigned as *Haloferax volcanii*. Samples from FB fumarole of 98°C displayed high abundance of Desulfurococcales (>90%) (exception of FBA1), with OTUs related to the marine hyperthermophilic *Pyrodictium* genus, followed by the orders Haloferacales (5%) and Nitrosopumilales (2%). FBA1 presented 96% of OTUs assigned as Haloferacales, and only 2% related to Desulfurococcales.

### Alpha-Diversity Estimates

**FIGURE 5:**
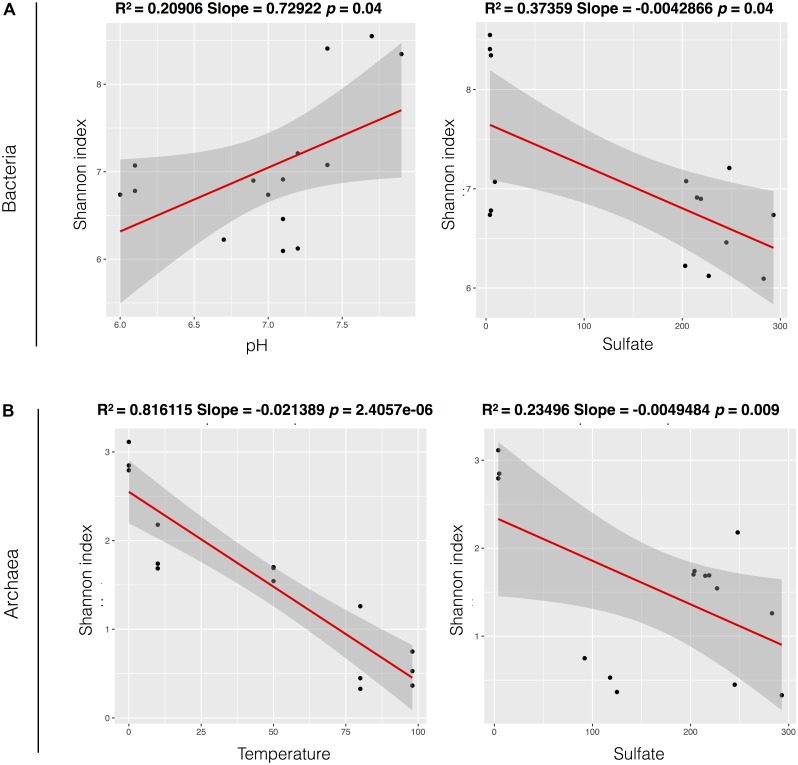
Models of univariate linear regression comparing relationship between alpha-diversity (Shannon index) and the parameters that were significant in the multiple linear regression models, for Bacteria **(A)** and Archaea domain **(B)**. The values of *p* and *R*^2^ are described above for each model.

Measures of bacterial and archaeal alpha-diversity were significantly higher in glaciers than in fumaroles (with exception of bacterial richness) (**Supplementary Table S2**). Univariate linear regressions were carried out to analyze relations between alpha-diversity (using Shannon index) and environmental parameters. Bacterial diversity was positively related with pH (*r*^2^ = 0.20, *p* = 0.049) and nitrate (*r*^2^ = 0.40, *p* = 0.006), and negative related with temperature (*r*^2^ = 0.38, *p* = 0.008), sulfate (*r*^2^ = 0.37, *p* = 0.009), and Na (*r*^2^ = 0.30, *p* = 0.01) (**Supplementary Figure S2**). Archaeal diversity was positively related with pH (*r*^2^ = 0.48, *p* = 0.002), ammonia (*r*^2^ = 0.22, *p* = 0.041), and nitrate (*r*^2^ = 0.33, *p* = 0.013), and negatively related with temperature (*r*^2^ = 0.81, *p* < 0.001) and sulfate (*r*^2^ = 0.23, *p* = 0.01) (**Supplementary Figure S3**). Although these *p*-values showed to be significant, only temperature exhibited a strong relation with archaeal diversity. Further, when we performed the multiple linear regressions, a combination of parameters showed strong correlations, and better explained bacterial (best multiple linear model with pH and sulfate parameters, *r*^2^ = 0.83), and archaeal alpha-diversity (best multiple linear model with temperature and sulfate, *r*^2^ = 0.86) (**Figure 5**).

### Bacterial Community Structure Across Environmental Parameters

Considering the geographic distance and remarkable differences in environmental conditions, 7 bacterial OTUs were shared among all sediment samples from Deception Island. Among these, three OTUs were related to Gammaproteobacteria (Methylophagaceae, Burkholderiaceae, and Nitrosomonadaceae), two related to Alphaproteobacteria (Rhizobiaceae and Rhodobacteraceae), and the other two were assigned as Acidimicrobiia (Ilumatobacteraceae) and Verrucomicrobiae (Rubritaleaceae). 184 bacterial OTUs were shared between fumaroles samples and 216 bacterial OTUs between glaciers samples, representing 3.1 and 3.7% of the total.

**FIGURE 6:**
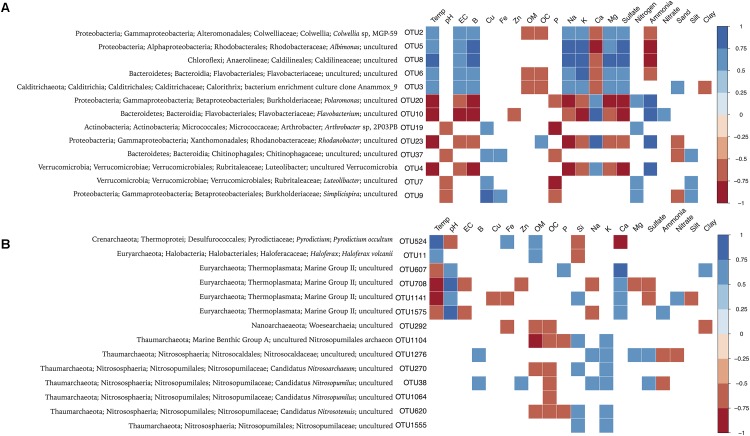
Spearman correlation between bacterial **(A)** and archaeal **(B)** abundant OTUs (>1% of relative abundance) and environmental parameters. Only parameters that exhibited *p* < 0.05 in correlations analysis are represented. The environmental parameters are: Temp (temperature), pH, EC (electrical conductivity), B, Cu, Fe, Zn, OM (organic matter), OC (organic carbon), P, Si, Na, K, Ca, Mg, sulfate, nitrogen, ammonia, nitrate, sand, silt, and clay.

In order to identify key environmental drivers of microbial composition, Spearman correlations were calculated, and only significant (*p* < 0.05) and strong correlations (*r* > -0.6 or 0.6) were considered. In general, bacterial OTUs prevalent in glaciers revealed negative correlations with marine and volcanic geochemicals (as electrical conductivity, Na, Mg, and sulfate), and positive correlations with ammonia (**Figure 6A**). In contrast, bacterial OTUs prevalent in fumaroles showed negative correlations with organic matter, organic carbon, Ca, and ammonia, and positive correlations with marine and volcanic geochemicals (as electrical conductivity, Na, K, Mg, sulfate, and temperature).

**FIGURE 7:**
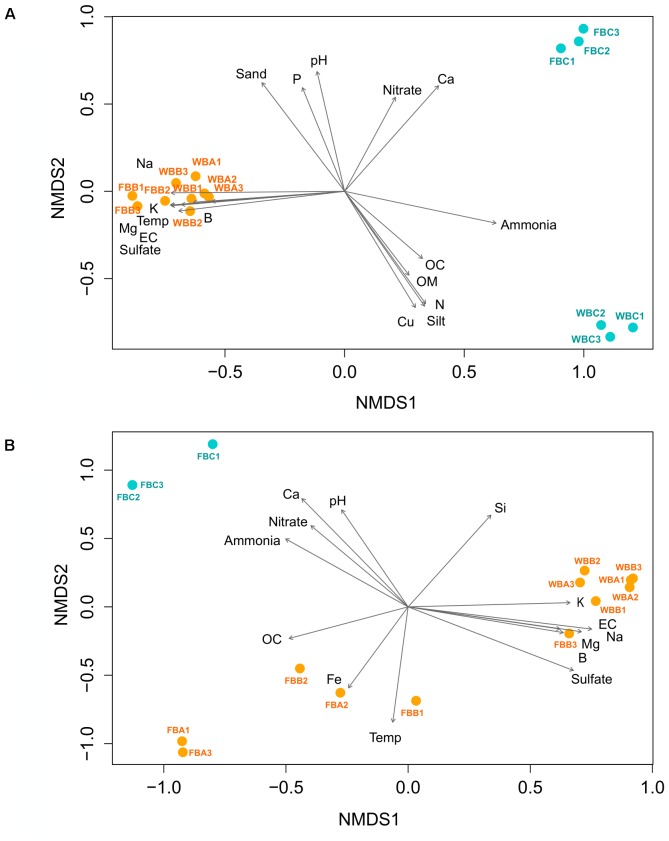
Non-metric multidimensional scaling (nMDS) ordination based on weighted UNIFRAC distance, with plotting of the environmental parameters for bacterial **(A)** and archaeal **(B)** communities. Each arrow is significantly correlated to the ordination (*envfit*, *p* < 0.05) and represents the direction and strength of the environmental parameter (Temp, temperature; EC, electrical conductivity; OC, organic carbon; OM, organic matter; N, nitrogen). Stress values = 0.007 **(A)** and 0.01 **(B)**.

Bacterial beta-diversity explored by Bray–Curtis and weighted Unifrac distances revealed a clear distinction between fumarole and glacier samples (**Figure 7A** and **Supplementary Figure S1a**). Adonis analysis showed that samples differed significantly when comparing fumaroles and glaciers (*p* = 0.001, *r*^2^ = 0.57), but not for geographic location (FB vs. WB) (*p* = 0.103, *r*^2^ = 0.16). Marine and volcanic geochemicals were positively correlated (*p* < 0.05) with fumarole bacterial communities in the nMDS analysis: temperature, Na, K, B, Mg, electrical conductivity, and sulfate (**Figure 5A**). In contrast, bacterial communities in WB glacier samples were more influenced by ammonia, total nitrogen, organic matter, organic carbon, Cu, and silt, whereas communities in FB glacier samples were positively related with nitrate and Ca.

### Archaeal Community Structure Across Environmental Parameters

Only one archaeal OTU (OTU38, related to Candidatus *Nitrosopumilus*) was shared between fumarole and glacier samples. Five OTUs were shared between fumarole samples, all related to Nitrosopumilales. Glaciers shared 14 OTUs, 4 related to Marine Group II (Thermoplasmata class) and the other 10 were not classified at phylum level.

Fewer environmental parameters exhibited strong correlations with archaeal composition, when compared to Bacteria (**Figure 6B**). In general, parameters that showed significant and higher correlation values were temperature, pH, and Ca. Glaciers-abundant OTUs, related to Marine Group II (Thermoplasmata class), showed positive correlations with pH, Ca, nitrate, and ammonia, and negative correlations with electrical conductivity, Na, sulfate, and temperature. *Haloferax* (OTU11) and hyperthermophilic Archaea *Pyrodictium* (OTU524) were positively correlated with temperature and Fe. OTUs highly abundant in WB fumaroles, mainly related to *Nitrosopumilus*, revealed positive correlations with Si, Na, K, and sulfate. Unlike for other archaeal OTUs, *Nitrosopumilus*-related OTUs showed no significant correlations with temperature.

Archaeal beta-diversity revealed a clear distinction between fumaroles and glaciers (**Figure 7B** and **Supplementary Figure S1b**). However, similar to what was observed for taxonomic composition, archaeal beta-diversity showed a distinct pattern between samples for the highest temperature fumaroles from FB. Adonis analysis based on Unifrac distance showed that archaeal communities were significantly distinct between fumaroles and glaciers (*r*^2^ = 0.49, *p* = 0.002), and likewise by their geographic locations (FB vs. WB) (*r*^2^ = 0.42, *p* = 0.003). nMDS revealed that temperature and Fe were the main parameters related to archaeal communities of high-temperature fumaroles (FB fumaroles), excepted for FBB3 (**Figure 7B**). Archaeal communities of WB fumaroles and FBB3 were positively influenced by sulfate, B, Mg, Na, K, and electrical conductivity, whereas FB glacier communities were positively influenced by nitrogen compounds (ammonia and nitrate), Ca, and pH.

## Discussion

It is well known that temperature and geochemical composition can act as strong selective pressures on microbial growth and survival (e.g., Sharp et al., 2014; Antranikian et al., 2017; Price and Giovannelli, 2017; Ward et al., 2017), but little is known about how volcanic activity shapes microbial community structure in polar ecosystems. Using high-throughput sequencing of 16S rRNA, we observed that steep gradients of temperature, salinity, and geochemical characteristics over a short distance (ca. 15 m) strongly influenced the microbial community structure and diversity of glaciers and fumaroles. To the best of our knowledge, this work represents the first study using deep DNA sequencing to characterize bacterial and archaeal communities in a polar marine volcano in Antarctica.

Despite these strong gradients, we detected members of some bacterial groups in all of our samples, both glacier and fumarole. These included members of the Proteobacteria, Planctomycetes, and Bacteroidetes phyla, highlighting the diversity and versatility of these groups. This observation is consistent with previous reports that members of these phyla can be detected in geothermal sites across temperatures ranging from 7.5 to 99°C (Sharp et al., 2014). We did not observe any archaeal phyla common to all of our samples, suggesting that members of archaeal phyla may have much narrower ecological niches than Bacteria at our sites.

Higher alpha-diversity was observed for both Bacteria and Archaea in glacier samples when compared to fumarole samples. This may be due to seasonal variations of temperature and nutrients in glaciers, which may select different groups of microorganisms and enhance microbial diversity in cold Antarctic ecosystems (Hopkins et al., 2006; Kirchman et al., 2014; Bowman et al., 2017; Franco et al., 2017). Indeed, we identified phylogenetically and functionally distinct groups in our glacier samples, as psychrophilic (*Flavobacterium*, *Luteolibacter*, *Rhodoferax*, *Polaromonas*, and *Arthrobacter*), methylotrophic (*Methylotenera*), denitrifying (*Rhodanobacter*), and nitrifying (*Nitrospira*, *Nitrosovibrio*) Bacteria.

The drivers of community diversity varied among the glacier, cooler fumaroles (<50°C) and the hotter fumarole (98°C) samples. The concentration of nitrogen compounds was strongly associated with microbial diversity in glaciers, while temperature, and the concentration of volcanic and marine geochemicals, was associated with diversity in cooler fumaroles. We observed that temperature was the main driver of diversity in the hotter fumarole, which was dominated by Archaea.

Consistent with these observations, we found that sequences associated with Nitrospirae were particularly common in our glacier samples. Nitrospirae are known as the most abundant and diverse group of Bacteria performing nitrification (Lücker et al., 2010). Their presence is understandable given the high concentration of nitrogen compounds we observed in our glacier samples. Further, previous reports have also shown high annual nitrogen fluxes in polar glaciers and the abundance of microorganisms related to nitrogen cycle (Segawa et al., 2014; Lutz et al., 2017). The presence of other abundant taxa, notably members of the phyla Verrucomicrobia and Patescibacteria (Parcubacteria, previously assigned as OD1), has been previously related to environments with high methane concentrations (Dunfield et al., 2007; Peura et al., 2012). Although their role in the methane cycle remains unclear, their co-occurrence with possibly methanogenic Archaea (*Methanomassiliicoccus*) in our FB glacier samples provides additional indication of their potential role in the methane cycle. Previous surveys in subglacial sediments (Wanda Glacier, King George Island) also detected members of the Methanomassilliicoccales (Pessi et al., 2015). Taken together, our observations suggest that the nitrogen and methane cycles may be central biogeochemical processes in Deception glaciers.

In contrast with glaciers, one of the most abundant bacterial phyla exclusively found in fumaroles was Calditrichaeota (previously assigned as *Caldithrix* within Deferribacteres phylum), a group identified in marine hydrothermal vents, such as those in Japan, Greece, and Mid-Atlantic ridge (Miroshnichenko et al., 2003, 2010; Takaki et al., 2010), and rarely described in Antarctic ecosystems. The majority of Calditrichaeota members described to date are thermophilic, with optimal growth temperatures between 40 and 65°C (Miroshnichenko et al., 2010). Gammaproteobacterial *Thalassomonas*, which have been mainly reported in tropical and coastal marine environments (Bowman and McMeekin, 2005), was the most abundant classified genus in our fumarole samples, and it was not found in our glacier samples. Other studies have detected these bacteria in deep sediments of the Gerlache Strait, Antarctica (López-García et al., 2001), and in shallow-sea hydrothermal vents on Panarea Island, Italy (Lentini et al., 2014).

Archaeal communities in our cooler fumarole samples (<50°C) were predominantly composed of the Nitrosopumilales and Nitrosocaldales orders, whose members are involved in the nitrogen cycle, particularly chemolithotrophic ammonia oxidation (Könneke et al., 2005; De la Torre et al., 2008). In environments without sources of organic energy and sunlight, ammonia oxidation contributes to primary productivity, explaining the success of marine members of Nitrosopumilales in ecological niches such as the deep-ocean and shallow polar waters during summer and winter (Könneke et al., 2005; Signori et al., 2014; Learman et al., 2016). Nitrosopumilales were previously reported in several Antarctic sediments, associated with Wanda Glacier, Weddell Sea, and along the west coast (Gillan and Danis, 2007; Pessi et al., 2015; Learman et al., 2016). Nitrosocaldales members are thermophilic, growing at higher temperatures (>60°C) than other thaumarchaeal ammonia oxidizers, and are globally distributed in various geothermal environments (De la Torre et al., 2008; Qin et al., 2017).

In our samples from the hotter fumarole (98°C), microbial communities were strictly composed of Archaea, in agreement with the current knowledge of hyperthermophilic growth on this temperature (e.g., Stetter, 2006; Stetter, 2013). These communities were not as diverse as those in cooler fumaroles, suggesting that increased temperature resulted in a decrease of microbial diversity, as previously reported (Sharp et al., 2014; Antranikian et al., 2017). The dominant genus in these sample (>80%) was the hyperthermophilic *Pyrodictium* (in particular in samples FBA2 and FBA3), which has never been previously reported in Antarctic ecosystems. Members of this genus are adapted to a wide range of temperatures, with optimum growth between 80 and 105°C (Stetter et al., 1983), and have been reported from shallow-sea hydrothermal vents, such as those in Vulcano, Italy (Stetter et al., 1983) and in Tachibana Bay, Japan (Takai and Sako, 1999), to deep-sea vents, as the Mariana Volcanic Arc (Nakagawa et al., 2006) and the Manus Basin, New Guinea (Takai et al., 2001). In addition, our work suggests that these marine hyperthermophiles could also colonize sediments associated to a non-submerged fumarole. Our results indicate that not only the marine influence, but also the local geochemistry and high temperature in the Deception hotter fumarole can act together as strong selective pressures and preferably select marine hyperthermophiles, despite the geographic isolation of Antarctica and its predominantly cold habitats. To date, hyperthermophilic Archaea have not been reported from any other geothermal site in Antarctica, probably due to the lower temperatures of continental volcanoes (up to 65°C at surface) (Herbold et al., 2014b), which may not select for these microorganisms.

Surprisingly, microorganisms related to halophilic *Haloferax* were identified in some of our fumarole samples with >80°C (FBA1, FBB1, and FBB2). Although previously described in Antarctica as abundant members of hypersaline subglacial lakes, such as the Deep Lake in Vestfold Hills (Williams et al., 2014), the high relative abundance of *Haloferax* in FB fumaroles (>80°C) was unexpected, since no hyperthermophilic members of this genus had been previously described (only thermophiles such as *H. volcanii*, with growth temperature <50°C) (Hartman et al., 2010). Possibly, *Haloferax* may have been collected in an inactive or even dead state or, less likely, undescribed members may tolerate these high temperatures through an unknown mechanism. Further, differences found in archaeal composition within triplicates of FB fumaroles may be related with the rapidly heat lost in higher temperature sites, which promote very accentuated temperature gradients and, consequently, can favor the presence of different micro-niches (Cao et al., 2012).

When compared to continental geothermal systems in Antarctica, such as Tramway Ridge in Mount Erebus (Soo et al., 2009; Herbold et al., 2014a), Deception fumaroles shared only few common taxa, notably Chloroflexi, Planctomycetes, and Nitrosopumilales. Bacterial groups previously detected in several geothermal fields and in shallow and deep-sea hydrothermal systems, such as Aquificae and Thermotogae phyla (e.g., Lebedinsky et al., 2007; Miller et al., 2009) and Epsilonproteobacteria class (now classified as Epsilonbacteraeota phylum) (e.g., Akerman et al., 2013; Anderson et al., 2017; Price and Giovannelli, 2017), were not identified in our samples. In addition, we detected different hyperthermophiles in our samples than those described by a previous study in Deception (Amenábar et al., 2013), possibly due to the use of different molecular tools and PCR primers in our study. Our results indicate the importance of future studies on microbial community of Deception Island using other molecular techniques such as metagenomics and metatranscriptomics to elucidate the functionality of extremophiles in polar marine volcanoes.

## Conclusion

By using a combination of 16S rRNA gene sequencing and physicochemical measurements we have found a strong separation of microbial community composition across environmental gradients, suggesting that bacterial community structure on Deception Island is strongly niche driven through the interaction of multiple environmental parameters (temperature, pH, salinity, sulfate, and nitrogen compounds), whereas archaeal community structure is mainly determined by temperature. Another important outcome of this study is the observation that Deception Island hosts bacterial and archaeal taxa previously reported from several highly contrasting environments, such as continental Antarctic volcanoes, non-volcanic polar ecosystems, and deep and shallow-sea hydrothermal vents. This likely reflects a mosaic of environmental conditions created by the interactions between volcanic activity, the marine environment, and the cryosphere, that can simultaneously select different groups of extremophiles (halophiles, psychrophiles, hyperthermophiles, and thermophiles). All of these factors make Deception a peculiar “open-air” laboratory to elucidate central questions regarding molecular adaptability, microbial evolution, and biogeography of extremophiles in polar regions.

## Author Contributions

AB collected the samples, conceived and designed the experiments, performed the experiments, analyzed the data, wrote the paper, and prepared figures and/or tables. CS analyzed the data, wrote the paper, prepared figures and/or tables, and reviewed drafts of the paper. DF collected the samples, analyzed the data, wrote the paper, and prepared figures and/or tables. RD conceived and designed the experiments, wrote the paper, and reviewed drafts of the paper. BB suggested the statistical analysis, wrote the paper, and reviewed drafts of the paper. VP conceived and designed the experiments, contributed reagents/materials/analysis tools, and wrote and reviewed drafts of the paper.

## Conflict of Interest Statement

The authors declare that the research was conducted in the absence of any commercial or financial relationships that could be construed as a potential conflict of interest.

## Supplementary Material

FIGURE S1 UPGMA dendrogram based on Bray–Curtis distance matrix of fumaroles (orange) and glaciers (blue) samples for Bacteria **(a)** and **(b)** Archaea.

FIGURE S2 Models of linear regression between temperature, pH, ammonia, nitrate, sodium and sulfate parameters, and the Shannon diversity index for the Bacteria domain. The values of *p* and *R*^2^ are described above for each model.

FIGURE S3 Models of linear regression between temperature, pH, ammonia, nitrate, sodium and sulfate parameters, and the Shannon diversity index for the Archaea domain. The values of *p* and *R*^2^ are described above for each model.

TABLE S1 Physicochemical parameters of the sediments, including temperature, granulometry, electrical conductivity, micronutrients (B, Cu, Fe, Mn, and Zn), organic matter, organic carbon, pH, P, Si, Na, K, Ca, Mg, Al, total nitrogen, nitrate, ammonia, and sulfate.

TABLE S2 Alpha-diversity estimates (number of OTUs, Ace = abundance-based coverage estimator, and Shannon and Simpson index) for Bacteria and Archaea. Results of Student’s *t*-test, testing whether means of “fumaroles” vs. “glaciers” samples are different. *P*-value < 0.05 is significant.
